# Deep Learning Applications in Computed Tomography Images for Pulmonary Nodule Detection and Diagnosis: A Review

**DOI:** 10.3390/diagnostics12020298

**Published:** 2022-01-25

**Authors:** Rui Li, Chuda Xiao, Yongzhi Huang, Haseeb Hassan, Bingding Huang

**Affiliations:** 1 College of Big Data and Internet, Shenzhen Technology University, Shenzhen 518118, China; lirui@sztu.edu.cn (R.L.); 2070416011@stumail.sztu.edu.cn (C.X.); 2060411015@stumail.sztu.edu.cn (Y.H.); haseeb@sztu.edu.cn (H.H.); 2 Guangdong Key Laboratory for Biomedical Measurements and Ultrasound Imaging, School of Biomedical Engineering, Shenzhen University Health Science Center, Shenzhen 518060, China; 3 College of Applied Sciences, Shenzhen University, Shenzhen 518060, China

**Keywords:** lung cancer, deep learning, lung nodule segmentation and classification, lung nodule computer-aided diagnosis

## Abstract

Lung cancer has one of the highest mortality rates of all cancers and poses a severe threat to people’s health. Therefore, diagnosing lung nodules at an early stage is crucial to improving patient survival rates. Numerous computer-aided diagnosis (CAD) systems have been developed to detect and classify such nodules in their early stages. Currently, CAD systems for pulmonary nodules comprise data acquisition, pre-processing, lung segmentation, nodule detection, false-positive reduction, segmentation, and classification. A number of review articles have considered various components of such systems, but this review focuses on segmentation and classification parts. Specifically, categorizing segmentation parts based on lung nodule type and network architectures, i.e., general neural network and multiview convolution neural network (CNN) architecture. Moreover, this work organizes related literature for classification of parts based on nodule or non-nodule and benign or malignant. The essential CT lung datasets and evaluation metrics used in the detection and diagnosis of lung nodules have been systematically summarized as well. Thus, this review provides a baseline understanding of the topic for interested readers.

## 1. Introduction

Lung cancer is one of the deadliest forms of cancer worldwide and represents a significant threat to human health and life. Over the past 50 years, the incidence and mortality rate of lung cancer have increased significantly in many countries. For example, according to the American Cancer Society, approximately 1,898,160 new cases will be diagnosed in 2021 and approximately 608,570 patients will die.

In general, the detection of lung cancer begins with the diagnosis of lung nodules, which are a leading radiological indicator for early diagnosis. The degree of malignancy of the nodule depends on its diameter. In most cases, nodules are small, rounded opacities within the pulmonary interstitium [1], which is a collection of support tissues within the lung that includes the alveolar epithelium, pulmonary capillary endothelium, basement membrane, and perivascular and perilymphatic tissues [2]. Lung nodules vary widely in terms of their shapes, sizes, and types [3]. Some nodules are spherical, with diameters from <2 mm to 30 mm [4], whilst other nodules have complex vascular attachments located in regions with large vessels and are challenging to detect. For instance, solid nodules (SN) and sub-solid nodules (SSNs) have densities only slightly above that of the surrounding lung parenchyma [5]. SNs are the most common type of nodule and contain the core functional lung tissues, whereas SSNs are pulmonary tumors with restricted ground-glass opacity (GGO). SSNs can be further categorized into part-solid nodules and pure ground-glass nodules [6]. These nodules show opacifications of a greater density than the nearby tissues and do not obscure underlying broncho-vascular structures [7]. 

As nodule size is related to malignancy, accurately measuring the diameter of nodules is critical to diagnosis. Several studies [1,8,9] have provided guidelines to this end. For example, the End-Use Load and Consumer Assessment Program (ELCAP) database [3] suggests a 1% malignancy rate for nodules smaller than 5 mm in diameter, 24% for nodules between 6 mm and 10 mm, 33% for nodules between 11 mm and 20 mm, and 80% for nodules of more than 20 mm [10]. However, errors may arise while measuring the diameter of very small nodules.

The treatment of lung cancer nodules is relatively complex. Nearly 70% of lung cancer patients require radiation therapy as part of their treatment, but such therapy can cause radiation-induced lung injury, which is a limiting toxicity that may decrease cure rates and increase morbidity and mortality. To overcome the defect of extracting additional information from nodules and to improve the accuracy of the classification of nodules, computer-aided diagnostic (CAD) systems are critical tools for radiologists. CAD systems are designed to overcome observational errors, reduce false-negative rates [11], and provide a second opinion for medical image interpretation and diagnosis [12]. Several studies have suggested that incorporating a CAD system into the diagnostic process can improve the performance of image diagnosis by decreasing inter-observer variation [13]. Likewise, CAD systems: provide quantitative support for clinical decisions such as biopsy recommendations [14]; assist in the performance of diagnostic checkups; reduce unnecessary false-positive biopsies [15] and thoracotomies [12]; and can be used to differentiate between the malignancy and benignancy of tumors [16,17].

Positive results in clinical studies have led to an upsurge in lung cancer detection using CAD models. Adopting and using such systems can improve survival rates through the diagnosis of lung nodules at early stages. The current computed tomography (CT) CAD applications search for pulmonary densities with specific physical characteristics (e.g., sphericity) that are representative of lung nodules [11]. Thus, CT CAD applications for lung nodule screening have become an active area of research. 

Initially, lung nodule diagnosis was heavily dependent on approaches that did not incorporate machine learning [18,19,20,21,22,23,24]. Later, machine learning-based approaches [25,26,27,28,29,30] were introduced to build the optimal boundary using data [31]. Recently, much research has been devoted to deep learning (DL)-inspired methods due to the accuracy of their predictions. DL-based models are different from conventional CAD systems, as they can be easily optimized and applied to a large amount of data [32]. DL relies on convolutional neural networks (CNNs) and has made a significant contribution to lung nodule diagnosis and management [33,34,35,36]. DL has been applied to pulmonary nodule diagnosis in three modules: nodule detection, segmentation, and classification. The detection module is responsible for localizing the nodule, the segmentation module aims to contour the nodule voxels, and the classification module predicts the nodule type (i.e., benign or malignant) [31]. 

Previous studies have conducted reviews of the research on pulmonary nodule detection techniques [31,32,37,38,39,40,41,42,43], with a range of objectives. However, the objective of this review article is to focus on the segmentation and classification modules of the pulmonary CAD system. The segmentation and classification tasks are the core components of a CAD system and ease the final decision-making regarding lung nodule, non-nodule, lung nodule type, and size. Furthermore, this work classifies the lung nodule segmentation literature based on different network architectures (general neural network and multiview CNN architecture), which will provide a clear understanding and intuition to a new researcher in the field for future research. For this purpose, the intended review study describes some recent and previous publications from reputable databases, including IEEE Xplore, Web of Science, PubMed, ScienceDirect, and Scopus, that have addressed the difficulties involved in diagnosing lung nodules. 

The rest of the paper is organized as follows: Section 2 provides an overview of a CAD framework; Section 3 and Section 4 describe the primary datasets and various evaluation metrics; and Section 5 and Section 6 outline the research on segmentation and classification approaches, respectively.

## 2. General CAD Framework for Detection and Diagnosis of Pulmonary Nodules

**Figure 1:**
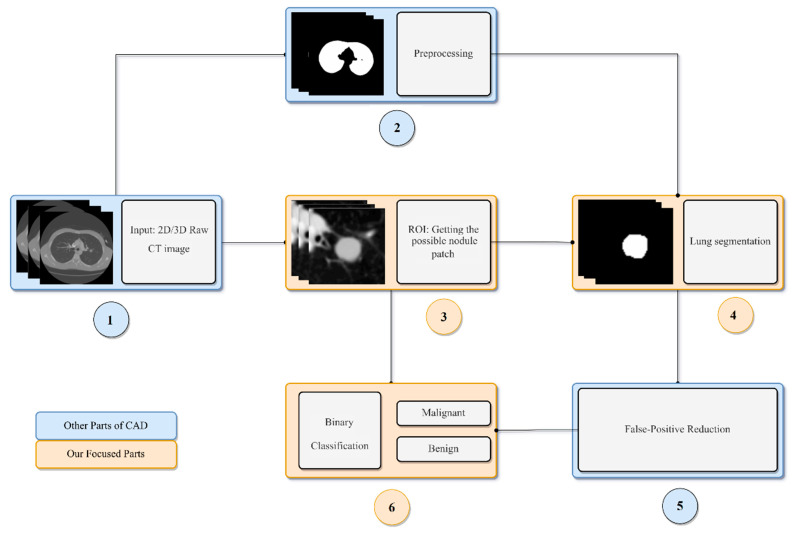
A general pipeline of a lung nodule CAD system.

Different CAD systems comprise different elements. The most common components of a CAD system are data acquisition, pre-processing, lung segmentation, lung nodule detection, false positive (FP) reduction, lung nodule segmentation, and lung nodule classification [42,44]. In the data acquisition step, the images used by the CAD system are collected. CT is a preferred choice for early nodule screening for this purpose, due to its high sensitivity and relatively low cost [45]. The pre-processing step involves the elimination of noise, artifacts, and other useless information from the images, thus improving the image quality for the subsequent steps. During lung segmentation, clinicians identify the boundaries of the lung from surrounding thoracic tissue in the CT images [46]. The detection step involves the localization of the lung nodule or mass and is followed by the FP reduction step [47]. The false-positive step is an essential process and involves identifying true lung nodules from the detected candidate nodules. In the lung nodule segmentation step, each nodule is segmented from the lung parenchyma. Then, in the feature extraction step, the characteristics of the nodule are quantified. These features are further used in the nodule classification step. Nodule classification is the final, and most vital, component of a CAD system and involves the differentiation of benign and malignant nodules. A typical pipeline of a lung nodule CAD system is depicted in Figure 1.

## 3. Datasets

DL models rely heavily on datasets. This is because the effective performance of the advanced learning algorithms is achieved using high-quality training datasets. However, high-quality, labeled training sets are usually complicated and expensive to produce. As a result, few public databases are available to support the development of lung nodule CAD systems. Indeed, whilst some organizations have made significant contributions to the formation of public datasets to facilitate research on the diagnosis of lung nodules using CT, these datasets do not adopt a standardized format for the storage of nodule information, and their labeling procedures also vary. For instance, some datasets are labeled using the coordinates of the vertices of the polygon, while other datasets are labeled according to the form of the center and radius of the nodule. A selection of databases used for lung nodule diagnosis research are briefly described below. 

LIDC-IDRI: The Lung Image Database Consortium and Image Database Resource Initiative (LIDC-IDRI) contains 1018 CT scans with marked-up annotated lesions in DICOM format. The diagnostic annotations are provided in XML format. For each CT scan, four experienced thoracic radiologists performed a two-stage annotation and revision procedure. In the first phase, the radiologists independently assessed each CT scan and labeled lesions as “nodule ≥ 3 mm,” “nodule = 3 mm,” or “non-nodule ≥ 3 mm”. After that, each radiologist independently reviewed the labels of the other lesions and gave their final diagnosis [48].

LUNA16: The extensive Lung Nodule Analysis 16 (LUNA 16) dataset is derived from LIDC-IDRI. It includes 36,378 annotations by radiologists on 888 selected CT scans. The authors only considered annotations categorized as nodules that were ≥3 mm as relevant lesions; nodules that were <3 mm and non-nodule lesions were not regarded as relevant for lung cancer screening protocols. A total of 1186 nodules were considered to be positive examples (i.e., the lesions that the algorithms should detect). Other nodules, i.e., those with different diameters, were regarded as irrelevant findings, and the marks on such locations were not counted as either FPs or true positives; rather, the irrelevant findings were excluded from the evaluation altogether [49].

Ali Tianchi: This dataset was developed by the Ali Tianchi Medical AI Competition and includes information on the nodules of 1000 patients. All the nodules were marked and confirmed by three doctors, except for the nodules analyzed by pathology. Nodules of 5–10 mm account for 50% of the dataset, and nodules of 10–30 mm account for the remaining 50%. Information on the position and size of the marked nodules is stored in CSV format.

NSCLC: The Non-Small Cell Lung Cancer (NSCLC) dataset contains information from 211 patients and 1355 CT images, with the images of 144 patients being obtained from axial CT imaging using an automatic segmentation algorithm. All the obtained segmentations were reviewed by thoracic radiologists, each of whom had more than five years of experience. The nodule annotations are stored in AIM format [50].

ELCAP: The Early Lung Cancer Action Program (ELCAP) database consists of a dataset of 50 low-dose documented whole-lung CT scans for detection purposes. The CT scans were obtained in a single breath-hold with a 1.25 mm slice thickness. The dataset also provides the locations of nodules between 2 mm and 5 mm in diameter that were detected by a radiologist [3].

ANODE09: This dataset belongs to the 2009 Automated Nodule Detection Database, which contains 55 CT scans, each with a slice thickness of approximately 1.0 mm. However, in the dataset, only five scans of 512 × 512 pixels were annotated; the other 55 scans were left unmarked to test the models. The annotated scans comprise 39 nodules and 31 non-nodules [51].

Table 1 summarizes the cited datasets and their detailed composition.

## 4. Lung Nodule Evaluation Metrics

Existing research has recognized the critical role played by evaluation metrics, which are used to measure the performance of the developed diagnostic models. The models for the detection and classification of lung nodules are mostly assessed according to sensitivity (SEN), specificity (SPEC), accuracy (ACC), precision (PPV), F1-score, receiver operating characteristic (ROC) curve, free-response operating characteristic (FROC), and area under the ROC curve (AUC), but the competition performance metric (CPM) can also be used to assess their performance [27]. The various metrics used to evaluate the performance of lung cancer algorithms are summarized in Table 2. 

## 5. Lung Nodule Segmentation

The accurate segmentation of lung nodules is challenging due to their small size, especially at the edge of the lung and near the blood vessels. Lung nodule segmentation is relatively broad and varies in terms of architecture, image pre-processing, and training strategy [52]. For instance, some DL-based nodule segmentation approaches are based on multiview neural network architecture, whilst others are based on general neural network architecture. Approaches that are based on multiview neural network architecture consider multiple views of lung nodules and combine them as an input to neural networks. General neural network architecture, on the other hand, builds on traditional CNN networks by changing or adding some blocks. 

Thus, this section, categorizes the literature on segmentation into two key approaches to pulmonary nodule segmentation: general neural network architecture and multiview architecture (i.e., multiscale problems). Moreover, the type and shape of a lung nodule significantly affect the choice of segmentation method for nodule detection. Therefore, this section also focuses on the different segmentation methods that are proposed for different types and sizes of nodules.

### 5.1. General Neural Network Architecture

Most published studies combined the conventional convolution network (CNN) architecture with neural network blocks for lung nodule segmentation. U-Net and Fully Convolutional Neural Networks (FCN) architectures are two basic structures that are frequently used. Numerous works have shown that convolutional neural networks architecture can significantly improve the performance of lung segmentation [53,54,55,56,57,58,59,60], especially semantic segmentation networks such as FCN [61] and U-Net [62]. Such networks implement two key steps. First, the image feature maps are extracted using a down-sampling process to filter the unnecessary information, whilst the important information is retained. Second, the resulting feature maps are then amplified through an up-sampling process to achieve a higher-resolution display image. 

Inspired by these networks, many segmentation studies modified and fine-tuned their models by leveraging the basic CNN architecture or changing or adding blocks to that CNN architecture. Huang et al. [57] proposed a system with four major modules: candidate nodule detection with faster regional-CNN (R-CNN), candidate merging, FP reduction using a CNN, and nodule segmentation using a customized FCN. Their model was trained and validated on the LIDC-IDRI dataset and achieved an average of 0.793 DSC. Tong et al. [59] utilized the U-Net architecture to perform the lung nodule segmentation. Their proposed method improved network performance by combining the U-Net with a residual block. Moreover, the lung parenchyma was extracted using a morphological method and the image was cropped to 64 × 64 pixels as an input to their improved network. The proposed model was trained and validated on the LUNA16 dataset and achieved 0.736 DSC. Usman et al. [56] proposed a dynamic modification region of interest (ROI) algorithm. This approach used Deep Res-UNet as the foundation for locating the input lung nodule volumes and improving lung nodule segmentation. Their method was divided into two stages. In the first stage, the Deep Res-Net was used for training and predicting the axial axis of the CT images. The second stage then focused on the new ROI in the CT image and used the deep Res-UNet architecture for the coronal and sagittal axes to train the network. The second stage also integrated the prediction results from the first stage into the final 3D results. Ultimately, the proposed method achieved 87.55% average DSC, 91.62% SEN, and 88.24% PPV.

Zhao et al. [60] proposed the implementation of a patch-based 3D U-Net and contextual CNN to automatically segment and classify lung nodules. This process began with a 3D U-Net architecture being used to segment the lung nodules, before generative adversarial networks (GANs) [63] were used to enhance the 3D U-Ne, and, finally, the contextual CNN was used to reduce the lung nodule segmentation FPs and improve the benign and malignant classification. This method achieved good results in segmenting lung nodules and classifying nodule types. Kumar et al. [55] utilized V-Net [64] for their lung nodule segmentation model. The proposed architecture adopted a 3D CNN model, using only the convolutional layers and ignoring the pooling layers. This model was evaluated on the LUNA16 dataset and achieved a DSC of 0.9615. Pezzano et al. [65] proposed a lung nodule segmentation network that added Multiple Convolutional Layer (MCL) blocks to the U-Net. The proposed network architecture was also divided into two phases, as opposed to being an end-to-end network. In the first phase, the researcher- trained model obtained the initial results; then, in the second phase, a morphological method was used for post-processing in order to highlight the nodules at the lung edges. The proposed architecture was trained and validated on the LIDC-IDRI database. The model achieved an 85.9% sensitivity, a 76.7% IoU, and an 86.1% F1-score. Keetha et al. [54] proposed a resource-efficient U-Det architecture by integrating U-Net with Bi-FPN (implemented in Efficient-Det). The proposed network was trained and tested on the LUNA dataset and achieved an average DSC of 82.82%, an average SEN of 92.25%, and an average PPV of 78.92%.

### 5.2. Multiview CNN Architecture

**Figure 2:**
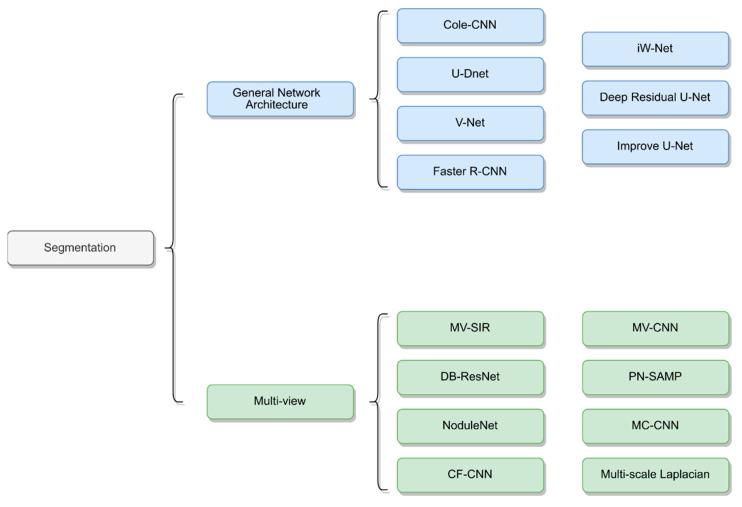
Network architecture of lung nodule segmentation.

Many research studies have proposed new architectures by taking multiple views of lung nodules as inputs to neural networks to achieve improved results [53,66,67,68,69,70]. These segmentation methods are primarily based on CNN networks and combine multiscale or multiview methods to train the neural networks. The structures of the different networks are depicted in Figure 2.

For instance, Zhang et al. [71] used a conventional method for nodule segmentation with a multiscale Laplacian of Gaussian filter to detect nodules. The proposed method was evaluated on the LUNA16 dataset and achieved a detection score of 0.947. Similarly, Shen et al. [70] considered different scales at feature levels in a single network and proposed the use of a multicrop CNN (MC-CNN) to automatically extract salient nodule information by employing a novel multicrop pooling strategy. Dong et al. [67] proposed a multiview secondary input residual (MV-SIR) CNN model for 3D lung nodule segmentation. Their approach achieved good results, with an 0.926 average DSC and 0.936 PPV.

Cao et al. [72] constructed a dual-branch residual network (DB-ResNet) and obtained improved lung segmentation results, with an average SEN of 89.35% and an average DSC of 82.74%. The proposed method employed two newly integrated schemes. First, it used the multiview and multiscale features of different nodules in CT images; second, it combined the intensity features with a CNN. Recently, Wu et al. [53] developed an interpretable, multitask learning CNN–joint learning for pulmonary nodule segmentation attributes and malignancy prediction (PN-SAMP) based on the U-Net architecture. The model achieved an average DSC of 73.89% and an average SEN of 97.58%. Finally, Wang et al. [69] proposed a central-focused CNN (CF-CNN) to segment lung nodules. Their architecture comprised two stages that used the same neural network to extract features and then merge those features. In addition, the authors used central pooling to preserve more of the features of the lung nodules. The model was tested on the LIDC-IDRI dataset and achieved an average DSC of 82.15% and an average SEN of 92.75%.

### 5.3. Segmentation Based on Lung Nodule Type

As mentioned above, pulmonary nodules have different types, shapes, and clinical features. Thus, the procedures used to detect nodules, as well as the associated challenges of such procedures, vary from case to case. In this section, we begin to address this issue by summarizing some of the works that have considered variations based on type. Table 3 shows the related methods and summarizes their key highlights.

Generally, lung nodules that are close to blood vessels and pleura are most challenging to detect. Thus, increasing the detail of the boundary nodules is core for all models. Pezzano et al. [65] proposed a network structure based on U-Net to segment lung nodules. The authors developed the multiple convolutional layers (MCL) module to fine-tune the details of the boundary nodules and post-process the nodule segmentation results. Morphological methods were used to strengthen the detail of the edge nodules. Dong et al. [67], meanwhile, proposed a model that incorporated features of voxel heterogeneity (VH) and shape heterogeneity (SH). VH reflects differences in gray voxel value, while SH reflects the characteristics of a better nodule shape. The authors found that VH can significantly learn gray information, whereas SH can better learn boundary information.

In addition, for juxta-pleural and small nodules, Cao et al. [72] proposed the DB-ResNet and presented a central intensity-pooling layer (CIP), which preserved the intensity features centered on the target voxel rather than the intensity information. For well-circumscribed nodules, Huang et al. [57] proposed a system that included segmentation and classification. In the segmentation step, the authors demonstrated that the model had a segmentation effect that was superior to that of other models.

However, the above methods exhibited slightly worse performance for the juxta-pleural, juxta-vascular, and ground-glass opacity nodules. Thus, to improve the performance of the model with respect to these types of nodules, AI-Shabi et al. [73] proposed the use of residual blocks with a 3 × 3 kernel size to extract local features and non-local blocks to extract global features. The proposed method managed to avoid many parameters and thus performed to a high standard. The LIDC-IDRI dataset was used for the training and testing. The proposed model achieved outstanding results as compared to DenseNet and ResNet in terms of transfer learning, scoring an AUC of 95.62%. Recently, Aresta et al. [58] constructed iW-Net, which comprised nodule segmentation and elements of user intervention. The proposed architecture performed well on large nodules without any user intervention. However, when user intervention was incorporated, the quality of the nodule segmentation in non-solid and sub-solid abnormalities improved significantly.

**Table 3:** Deep learning-based lung nodule segmentation architectures and their key information.

Study	Year	Architecture	Dataset	Approach	Performance
Pezzano et al. [65]	2021	CoLe-CNN	LIDC-IDRI	2D Based U-Net Inception-v4 architecture Mean Square Error function	F1 = 86.1 IoU = 76.6
Dong et al. [67]	2020	MV-SIR	LIDC-IDRI	2D/3D Residual block Secondary input Multi views Voxel heterogeneity (VH) Shape heterogeneity (SH)	ASD = 7.2 ± 3.3 HSD = 129.3 ± 53.3 DSC = 92.6 ± 3.5 PPV = 93.6 ± 2.2 SEN = 98.1 ± 11.3
Keetha et al. [54]	2020	U-DNet	LUNA16	2D Based U-Net Bi-FPN Efficient-Det Mish activity function	DSC = 82.82 ± 11.71 SEN = 92.24 ± 14.14 PPV = 78.92 ± 17.52
Cao et al. [72]	2020	DB-ResNet	LIDC-IDRI	2D/3D ResNet CIP Multiview Multiscale Central Intensity-Pooling	DSC = 82.74 ± 10.19 ASD = 19 ± 21 SEN = 89.35 ± 11.79 PPV = 79.64 ± 13.34
Kumar el al. [55]	2020	V-Net	LUNA16	3D V-Net PReLU Only fully convolutional lays	DSC = 96.15
Usman et al. [56]	2020	Adaptive ROI with Multi-view Residual Learning	LIDC-IDRI	2D/3D the Deep Residual U-Net Adaptive ROI Multiview	SEN = 91.62 PPV = 88.24 DSC = 87.55
Tang et al. [74]	2019	NoduleNet	LIDC-IDRI	3D Multitask Residual-block Detection, FPR, segmentation Different loss function	DSC = 83.10 CPM = 87.27
Huang et al. [57]	2019	Faster R-CNN	LUNA16	2D Faster RCNN Merge overlap FP reduction Based FCN	ACC = 91.4 DSC = 79.3
Aresta et al. [58]	2019	iW-Net	LIDC-IDRI	3D Based U-Net two points in the nodule boundary none heavy pre-processing steps augmentation	IoU = 55
Hesamian et al. [75]	2019	Atrous convolution	LIDC-IDRI	2D Atrous convolution Residual Network Weight loss Normalize to 0, 255	DSC = 81.24 Precision = 79.75
Liu et al. [76]	2018	Mask R-CNN	LIDC-IDRI	2D Backbone: ResNet101, FPN transfer learning RPN FCN	73.34 mAP 79.65 mAP
Khosravan et al. [77]	2018	Semi-supervised multitask learning	LUNA16	3D Data augmentation Semi-supervised FP reduction	SEN = 98 DSC = 91
Wu et al. [53]	2018	PN-SAMP	LIDC-IDRI	3D 3D U-Net WW/WC Dice coefficient loss Segmentation, classification	DSC = 73.98
Tong et al. [59]	2018	Improved U-NET network	LUNA16	2D U-Net Modify residual block Obtain lung parenchyma	DSC = 73.6
Zhao et al. [60]	2018	3D U-Net and Contextual Convolutional Neural Network	LIDC-IDRI	3D 3D U-Net GAN Morphological methods Residual block Inception structure	None
Wang et al. [66]	2017	MV-CNN	LIDC-IDRI	2D/3D Mutilview A multiscale patch strategy	SEN = 83.72 PPV = 77.59 DSC = 77.67
Wang et al. [69]	2017	CF-CNN	LIDC-IDRI/GDGH	2D/3D Central pooling 3D patch 2D views A sampling method Two datasets	LIDC: DSC = 82.15 ± 10.76 SEN = 92.75 ± 12.83 PPV = 75.84 ± 13.14 GDGH: DSC = 80.02 ± 11.09 SEN = 83.19 ± 15.22 PPV = 79.30 ± 12.09

## 6. Classification

In addition to the above works on nodule detection, other studies have focused on the classification of candidate nodules according to the following categories: benign, primary cancer, and metastatic cancer. Classification tasks also incorporate FP screening or reduction, which can improve the accuracy of classification. Therefore, nodule classification is essential because it assists doctors in diagnosing benign and malignant nodules, thus improving the overall efficiency of diagnoses. For this purpose, deep neural networks are used to analyze various characteristics of lung nodules, such as their shape and location. Neural networks are further used to analyze the input lesion area and to predict the final results. This section therefore provides a brief overview of the existing literature to illustrate the various techniques that have been developed to support malignancy detection and the classification of lung nodules.

### 6.1. Classification as Nodule or Non-Nodule

When searching for candidate nodules, the presence of blood vessels and other soft tissues can lead to the detection of FPs. Adopting effective classification techniques can reduce FP detection and, thus, significantly improve the accuracy of nodule identification and reduce the difficulty of subsequent tasks. For example, Wu et al. [78] developed a deep residual network to classify lung nodules. For this, the authors adopted a transfer-learning-based 50-layer Res-Net structure with global average pooling. The Principal Component Analysis (PCA) technique was used to reduce the feature size and number of parameters. The architecture was trained and tested on the LIDC-IDRI dataset. It achieved an accuracy of 98.23%, a sensitivity of 97.7%, a specificity of 98.35%, and an F1-score of 98.06%. Tran et al. [79] proposed an LdcNet model to improve the accuracy of the classification of pulmonary nodules. They used a 15-layer 2D CNN and employed Focal loss to classify the pulmonary candidates as nodule or non-nodule. They also used data extracted from the LIDC-IDRI and LUNA16 data sets. Their model achieved an accuracy of 97.2%, a sensitivity of 96.0%, and a specificity of 97.3%.

Li et al. [80] used CNN and a fully connected layer to classify pulmonary nodules. They divided the input patch into 32 × 32 and 64 × 64 sections using the nodule size and trained two identical networks. The proposed network was trained on 62,492 ROI samples, including 40,772 nodules and 21,720 non-nodules, from the LIDC-IDRI dataset. It achieved an accuracy of 86.4% and a sensitivity of 89.0%. Mastouri et al. [81] proposed a network to classify lung nodules, known as bilinear CNN (BCNN). Their network used VGG-16 and VGG-19 as the training network to extract the relevant features, while an SVM classifier was utilized for the nodule classification. The additional ablation experiments conducted by the authors on the LIDC-IDRI dataset demonstrated that the SVM classifier performed better than soft-max, KNN, and other classifiers. Additionally, using VGG-16 + VGG-19 was shown to be superior to using a single network (e.g., VGG-16 + VGG-16). Ultimately, the proposed network achieved an accuracy of 91.99% and an AUC of 95.9%. Finally, Yang et al. [82] suggested using a two-stage CNN (2S-CNN) to classify the candidates into nodules/non-nodules, leading to a classification accuracy of 89.6%.

### 6.2. Classification as Benign or Malignant 

Given that lung nodules have various types and shapes, radiologists find it challenging to classify them as benign or malignant based on CT images—a topic that numerous studies have focused on. Table 4 summarizes the methods adopted in each study.

For example, Ali [83] proposed using a transferable texture CNN to improve the classification of pulmonary nodules in CT scans. The proposed approach used the LIDC-IDRI and LUNGx datasets for training and validation. Furthermore, a transfer learning technique was used to extract the weights of some layers of the training model to retrain the LUNGx dataset using the proposed architecture. The trained model achieved an accuracy of 97.69%, an error rate of 3.3%, an AUC of 99.11%, and a sensitivity of 97.19% on the LIDC-IDRI dataset. It achieved excellent results, with a 90.91% accuracy, 91.37% sensitivity, and 94.14% AUC on the LUNGx dataset.

Al-Shabi et al. [73] proposed combining a deep local-global network with residual and non-local blocks to extract the global features with few parameters. Their proposed network successfully analyzed the shape and size of the nodules. The architecture training and testing were performed using the LIDC-IDRI dataset. Their results produced an AUC of 95.62%, an accuracy of 88.46%, a sensitivity of 88.66%, and a precision of 87.38%. In another study [84], also led by Al-Shabi, gated dilated networks were used to classify nodules as malignant or benign. The proposed network incorporated a context-aware sub-network that analyzed the input features and guided the features to a suitably dilated convolution to reduce the parameters. The framework achieved better recognition of medium-sized nodules, based on an evaluation using the LIDC-IDRI dataset. Similarly, another model, MoDenseNet [85], used a two-pathway 3D CNN architecture with dense blocks to classify nodules as malignant or benign. Patches of the same nodule at different scales (e.g., 50 × 50 × 5, 100 × 100 × 10) were used as the network’s inputs. Finally, the intermediate and final feature maps were concatenated and classified into the prediction results. The proposed model was trained on 686 lung nodules from the LIDC-IDRI dataset. The testing results produced a TPR of 90.47%, a TNR of 90.33%, a PPV of 90.55%, an AUC of 95.48%, and an accuracy of 90.40%. 

Wu et al. [53] developed a classification network to concatenate the feature map of the third layer of their proposed lung nodule segmentation network with the feature map of the final result of the lung nodule segmentation network so as to classify nodules as benign or malignant. Their model was evaluated on the LIDC-IDRI dataset and achieved an accuracy of 89.33%. Zhao et al. [60] proposed using a contextual CNN to reduce FPs when classifying nodules as benign or malignant. Moreover, they concatenated the feature map of the contextual CNN with the feature map of the sixth layer of the CNN. The proposed framework performed the required classification accurately and efficiently. In another study, a three-pathway multiview CNN model [86] was used to classify lung nodules as benign or malignant, with a curriculum learning strategy also being adopted to enable the network to learn more precise weightings. The model was evaluated on the LIDC-IDRI dataset and achieved a sensitivity of 91.07%, a specificity of 88.64%, a precision of 89.35%, an accuracy of 89.90%, and an AUC of 94.59%. Liu et al. [87] developed an MV-CNN framework to classify pulmonary nodules. They used multichannel CT images to improve the extraction of feature information, incorporating seven patches at different scales, and proposed both binary and ternary classification. Furthermore, they performed multiple experiments to prove that multichannel input performed better than single-channel input and achieved better quantitative results compared with other models.

Shen et al. [70] proposed a multicrop pooling layer technique based on a spatial pyramid pooling network (SPPNet) that captured nodule-centric visual features without including a nodule classification step after segmentation. The proposed model was evaluated on the LIDC-IDRI dataset and achieved an accuracy of 87.14%, an AUC of 93%, a sensitivity of 77%, and a specificity of 93%. Other methods have also been proposed to classify lung nodules as benign or malignant. For example, one study proposed that an auto-encoder and binary decision tree be used [88]. For this, the fully connected (FC) layer was used to extract the features, before the binary decision tree was used to classify the nodules; after that, the LIDC-IDRI dataset was used to evaluate the model.

In another study [89], the authors developed an unsupervised method based on a deep sparse autoencoder (SAE) to extract the robust features from the lung. They then used a linear support vector machine (SVM) to classify the nodules. Nasrullah et al. [90] utilized a gradient boosting machine (GBM) and MixNet [91] to learn the complex features of nodules. Their approach combined the advantages of dual-path networks (DPN) with residual networks (ResNet) and Densly Connected Networks (DenseNet) to perform the lung nodule classification tasks. Akila et al. [92] proposed an efficient deep neural network for automatic pulmonary nodule classification with a supervised approach. Akila, therefore, employed several deep neural networks, including RNN, LSTM, and CNN, and produced results showing that RNN was not less suitable for learning patterns than LSTM and CNN models, with the CNN-based classifiers achieving a 25% higher accuracy than the RNN model. Similarly, another study [93] presented a novel interpretable deep hierarchical semantic CNN (HSCNN) for pulmonary nodule classification. The proposed model was trained and tested on the LIDC dataset and achieved better results than common 3D CNN approaches. Ge et al. [94] proposed a model with DenseNet-based architecture together with 3D filters and pooling kernels. The model achieved a classification accuracy of 92.4% on the LUNA16 dataset. Guobin [95] used a squeeze-and-excitation (SEN) network and aggregated residual transformations (SE-ResNeXt) to perform the required classification task. The model was evaluated on the LUNA16 dataset and achieved an accuracy of 91.67%. Yang [96] developed a novel two-stage CNN (2S-CNN) to classify lung CT images in an approach that consisted of two CNNs—one a basic network and the other a simplified version of GoogLeNet. The experimental results of this study produced an accuracy of 89.6%.

In another study [97], the authors proposed a multiscale synchronized deep supervision technique using an AlexNet network and synchronized deep supervision (SDS). The multiscale spatial pyramid strategy was used to extract the features from lung nodules at different scales. Another recent work [98] applied a 3D CNN (MMEL-3DCNN) to classify different types of lung nodules. The proposed approach was built on a multimodel network architecture and applied ensemble learning to improve the robustness of the nodule classification model. The experimental results were verified on the LIDC-IDRI and the model obtained satisfactory classification results. Kai [99] first constructed a residual attention network (RAN) and SEN network to extract the spatial and contextual features of the nodules. Next, they introduced a novel multiscale attention network (MSAN) to capture the multiscale attention features and used a GBM algorithm to differentiate benign and malignant nodules. Experiments on the LIDC-IDRI database achieved an accuracy of 91.9%, a sensitivity of 91.3%, an FP rate of 8.0%, and an F1-score of 91.0%. The detailed results comparison for segmentation and classification models are summarized in Table 5 and Table 6.

**Table 4:** Overview of lung nodule classification architectures and their key information.

Year	Author	Method	Performance
2021	Ge Zhang [94]	3D DenseNet Dense block Transition layer Malignant or benign	ACC = 92.4% SEN = 87.0% SPEC = 96.0%
2020	Akila Agnes [92]	CNN 2D Malignant or benign	SEN = 81% SPEC = 91.9% Precision = 87.8% ACC = 87.26% AUC = 0.944
2020	Rekka Mastouri [81]	BCNN 3D VGG16 VGG19 SVM Nodule or non-Nodule	ACC = 91.99% SEN = 91.85% SPEC = 92.27% F1-score = 93.76% FPR = 7.72%
2020	Hong Liu [98]	MMEL-3DCNN VggNet ResNet InceptionNet Multinetwork Malignant or benign	SEN = 0.837% SPC = 0.939% ACC = 0.906% AUC = 0.939%
2020	Kai Xia [99]	Residual learning Dense learning MSAN GBM 3D attention Dual-path Malignant or benign	ACC = 91.9% SEN = 91.3% FP rate = 8.0% F1-score = 91.0%
2020	Wu et al. [78]	2D Migration learning ResNet50 PCA Nodule or non-nodule	ACC = 98.23% SEN = 97.7% SPEC = 98.35% F1 = 98.06% Precision = 98.64% FPR = 1.65%
2020	Ali et al. [83]	2D Energy Layer Transfer learning Malignant or benign	ACC = 96.69% ± 0.72% Error rate = 3.3% ± 0.72% AUC = 99.11% ± 0.45% SEN = 97.19% ± 0.57%
2019	Yang An [96]	2S-CNN Inception CNN Nodule or non-nodule	ACC = 89.6%
2019	Zhang Li [97]	AlexNet SDS MPPS Malignant or benign	ACC = 93.68% SEN = 95.17% SPEC = 93.92%
2019	Tran et al. [79]	2D Focal loss Nodule or non-nodule	ACC = 97.2% SEN = 96.0% SPEC = 97.3%
2019	Al-Shabi et al. [73]	2D Residual block Non-Local block Self-attention Malignant or benign	AUC = 95.62% ACC = 88.46% Precision = 87.38% SEN = 88.66%
2019	Al-Shabi et al. [84]	2D Multiple dilated convolutions Context-Aware sub-network Mid-range sized nodules Malignant or benign	AUC = 93.15% ACC = 92.57% Precision = 91.85% SEN = 92.21%
2019	Guobin Zhang [95]	SE-ResNeXt SENet ResNet Malignant or benign	AUC = 0.9563 ACC = 91.67%
2018	Shiwen Shen [93]	HSCNN 3D Sub-task Malignant or benign	AUC = 0.856 ACC = 0.842 SEN = 0.705 SPEC = 0.889
2018	Dey et al. [85]	3D Multiscale Multioutput Dense block Malignant or benign	TPR = 90.47% TNR = 90.33% PPV = 90.55% AUC = 95.48% ACC = 90.40%
2018	Wu et al. [53]	3D 3D U-Net WW/WC	ACC = 97.58%
2018	Zhao et al. [60]	3D CNN Inception structure	None
2017	Nibali et al. [86]	2D ResNet18 Transfer learning Curriculum learning Malignant or benign	SEN = 91.07% SPEC = 88.64 Precision = 89.35% AUC = 94.59% ACC = 89.90%
2017	Liu et al. [87]	2D Multiscale Multichannel Binary/Ternary classification Malignant or benign	SEN = 90.18% SPEC = 100% Error rate = 5.41% AUC = 0.981
2016	Li et al. [80]	2D CNN Multiscale Two networks Nodule or non-nodule	ACC = 86.4% SEN = 89.0%
2016	Shen et al. [70]	3D CNN Multi-crop pooling layer Malignant score	ACC = 87.14% AUC = 0.93 SEN = 0.77 SPEC = 0.93
2015	Kumar et al. [88]	2D CNN Binary decision tree Malignant or benign	ACC = 75.01% SEN = 83.35% FP = 0.39 FP/patient

## 7. Challenges and Future Perspectives

Despite recent breakthroughs in deep learning for diagnosing pulmonary lung nodules, a large volume of chest scan data has several challenges, including feature extraction, nodule detection, false-positive reduction, and benign-malignant classification [100]. For effective feature extraction and benign-malignant classification, recurrent neural networks (RNNs) [101], deep belief networks (DBNs) [102], and autoencoders [103] could be used. Similarly, advances in graphical processing units (GPUs) positively impact the use of deep learning. As a result of the parallelization of CNNs, better feature extraction and classification may be achieved [104].

The majority of the CAD system’s decision-making for nodule detection relies heavily on supervised learning approaches. However, supervised learning is costly and time-consuming because it relies on massive labeled datasets. Furthermore, a model trained on fewer data has a higher risk of overfitting and convergence problems. Unsupervised learning approaches such as transfer learning techniques may be more suited in such situations. Balanced datasets, such as those used by [105], could be used to limit false positives.

Aside from the challenges mentioned above, one of the most significant obstacles to deep learning pulmonary nodule analysis is the scarcity of well-annotated and large-scale labeled datasets. Therefore, high quality and large datasets are also pressing for setting up an efficient deep learning CAD system to detect pulmonary nodules. From this review study, it has been also noted that the leading solutions employed CNNs and used the provided set of nodule candidates.

In the future, substantial resources and strong regulatory criteria will be required for lung nodule screening in order to ensure significant advantages and attempt to reduce the number of false negatives and positives. Future research should also focus on developing and validating simpler nodule evaluation algorithms by incorporating emerging diagnostic modalities like molecular signatures, biomarkers, and liquid biopsies [106]. For this purpose, deep learning and machine learning algorithms will be a perfect choice that allows more accurate automatic characterization and classification of nodules with higher accuracy and could led to revolutionary changes in radiology.

## 8. Conclusions

Recent research has incorporated DL strategies, achieving promising results in relation to the detection of lung nodules using CT images. However, segmenting and classifying lung nodules for the purposes of detection and diagnosis remains challenging.

Many lung nodule segmentation approaches are based on either general or multiview neural network architecture. Most studies using multiview neural networks incorporated some new architecture by taking multiple views of the lung nodules and using those views as inputs to the neural networks. In contrast, the general neural network-based methods were primarily based on U-Net architecture. Likewise, different lung nodule segmentation methods were adopted for different types of lung nodules, including boundary, juxta-pleural, small, well-circumscribed, and large nodules.

In terms of classification methods, many techniques have been proposed for the classification of lung nodules (e.g., whether they are benign or malignant). Most of the conducted works focused on supervised, as opposed to semi-supervised, learning. It is noted that limited datasets are available for training and testing the models, with the LIDC-IDRI dataset being the most used. To compare different approaches and their results, a series of tables were arranged to summarize the important findings. Different models used different metrics to verify their results on a range of datasets. Overall, the best-performing models vary greatly according to their data type, annotation conditions, and experimental aims. Therefore, comparing their performance is not straightforward. In spite of this limitation, our analysis of the literature is quite successful in highlighting the importance of building robust DL architectures to segment and classify lung nodules accurately and efficiently. Last but not least, future research must be initiated in terms of new guidelines and computer-based algorithms that are easy to use, which would provide great aid to both researchers and medical practitioners.

## Figures and Tables

**Table 1:** Cited datasets and their composition.

Dataset	The Number of CT Scans	The Number of Nodules	Annotation
LIDC-IDRI	1018	36,378	√
LUNA16	888	13,799	√
Ali Tianchi	1000	1000	√
NSCLC	211	-	√
ELCAP	50	-	√
ANODE09	55 (only 5 CT scans)	39	√

**Table 2:** Various evaluation metrics used for lung cancer/nodule diagnosis.

Metric	Brief	Expression
Sensitivity (SEN)	Measures the proportion of positives that are correctly identified	$SEN=\frac{TP}{TP+FN}$
Accuracy (ACC)	Classification accuracy of the classifier	$ACC=\frac{TP+TN}{TP+FN+FP+TN}$
Positive predictive value (PPV)	The proportions of positive results in statistics and diagnostic tests that are truly positive	$PPV=\frac{TP}{TP+FP}$
Dice Similarity Coefficient (DSC)	A statistics used to gauge the similarity of two samples.	$DSC=\frac{2TP}{2TP+FP+FN}$
Intersection over Union (IoU)	The IoU measurement gives the similarity between the predicted area and the real area of the objects present in the set of images	$IoU=\frac{TP}{TP+FP+FN}$
F1-Score	Used in statistics to measure the accuracy of a binary classification model	$F1=\frac{2\left(SEN+PPV\right)}{\left(SEN+PPV\right)}$
Receiver Operating Characteristic (ROC)	A curve depicting the relationship between the sensitivity and specificity (Y-axis is TP rate and *X*-axis is the FP rate)	-
Free Receiver Operating Characteristic (FROC)	Similar to the ROC curve, differing only in the *X*-axis. The *X*-axis is the FP rate per image (or per scan)	-
Area Under Curve (AUC)	Total area under the ROC curve	-
Competition Performance Metric (CPM)	Average of the Sensitivity at seven defined FP rates in the FROC curve: 1/8,1/4,1/2,1,2,4,8 FPs/scan	-
Mean Average Precision (mAP)	Mean Average Precision	-

Note: *TP*: true positive; *TN*: true negative; *FN*: false negative; *FP*: false positive.

**Table 5:** Results comparison of nodule segmentation models.

Year	Author	Dataset	PPV (%)	SEN (%)	DSC (%)	IOU	Architecture	Approach
2020	Dong et al. [67]	LIDC-IDRI	93.6	98.10	92.6	-		Multiview
2020	Cao et al. [72]	LIDC-IDRI	79.64	89.35	82.74	-		Multiview
2017	Wang et al. [66]	LIDC-IDRI	77.59	83.72	77.67	-		Multiview
2017	Wang et al. [69]	LIDC-IDRI/GDGH	75.84	92.75	82.15	-		Multiview
2017	Shen et al. [70]	Random datasets	87.14	0.77	-	-	MC-CNN	Multiview
2021	Pezzano et al. [65]	LIDC-IDRI	-	-	-	76.6	Nodule type	General
2020	Keetha et al. [54]	LUNA16	78.92	92.24	82.82	-	U-Net et al.	General
2020	Kumar et al. [55]	LUNA16	-	-	96.15	-	U-Net et al.	General
2020	Usman et al. [56]	LIDC-IDRI	88.24	91.62	87.55	-		General
2019	Huang et al. [57]	LUNA16	-	-	79.3	-	U-Net et al.	General
2018	Wu et al. [53]	LIDC-IDRI	-	-	73.98	-	U-Net et al.	General
2018	Tong et al. [59]	LUNA16	-	-	73.6	-	U-Net et al.	General
2018	Zhao et al. [60]	LIDC-IDRI	-	-	-	-	U-Net et al.	General
2018	Liu et al. [76]	LIDC-IDRI	-	-	-	-	FCN	General
2019	Aresta et al. [58]	LIDC-IDRI	-	-	-	55		Nodule type
2019	Hesamian et al. [75]	LIDC-IDRI	-	-	81.24	-		-
2018	Khosravan et al. [77]	LUNA16	-	98	91	-		semi-supervised
2019	Tang et al. [74]	LIDC-IDRI	-	-	83.10	-		-

**Table 6:** Results comparison of nodule classification models.

Year	Reference	Dataset	SEN (%)	AUC (%)	ACC	Classification
2021	Ge Zhang [94]	LUNA16	87.00	-	92.40	MOB
2020	Akila Agnes [92]	LIDC- IDRI	81.00	94.40	-	MOB
2020	Hong Liu [98]	LIDC-IDRI	0.837	93.90	90.60	MOB
2020	Kai Xia [99]	LIDC-IDRI	91.30	-	91.90	MOB
2020	Ali et al. [83]	LIDC-IDRI	98.10	99.11	96.69	MOB
2019	Zhang Li [97]	LIDC-IDRI	95.17	-	93.68	MOB
2019	Guobin Zhang [95]	LUNA16	-	95.63	91.67	MOB
2019	Al-Shabi et al. [73]	LIDC-IDRI	88.66	95.62	88.46	MOB
2019	Al-Shabi et al. [84]	LIDC-IDRI	92.21	93.15	92.57	MOB
2018	Shiwen Shen [93]	LIDC-IDRI	0.705	0.856	0.842	MOB
2018	Dey et al. [85]	LIDC-IDRI/Themselves dataset	-	95.48	90.40	MOB
2017	Nibali et al. [86]	LIDC-IDRI	91.07	94.59	89.90	MOB
2016	Shen et al. [70]	LIDC-IDRI	77.00	93.00	87.14	MOB
2015	Kumar et al. [88]	LIDC-IDRI	83.35	-	75.01	MOB
2020	Wu et al. [78]	LIDC-IDRI	97.70	-	98.23	NON
2019	Yang An [96]	LIDC-IDRI	-	-	89.60	NON
2019	Tran et al. [79]	LIDC-IDRI	96.00	-	97.20	NON
2020	Rekka Mastouri [81]	LUNA16	91.85	-	91.99	NON
2018	Wu et al. [53]	LIDC-IDRI	-	-	97.58	NON
2018	Zhao et al. [60]	LIDC-IDRI	-	-	-	NON
2017	Liu et al. [87]	LIDC-IDRI	90.18	98.10	-	others
2016	Li et al. [80]	LIDC-IDRI	89.0	-	86.40	others

MOB: Malignant or benign; NON: nodule or non-nodule; Other: except MOB and NON.
